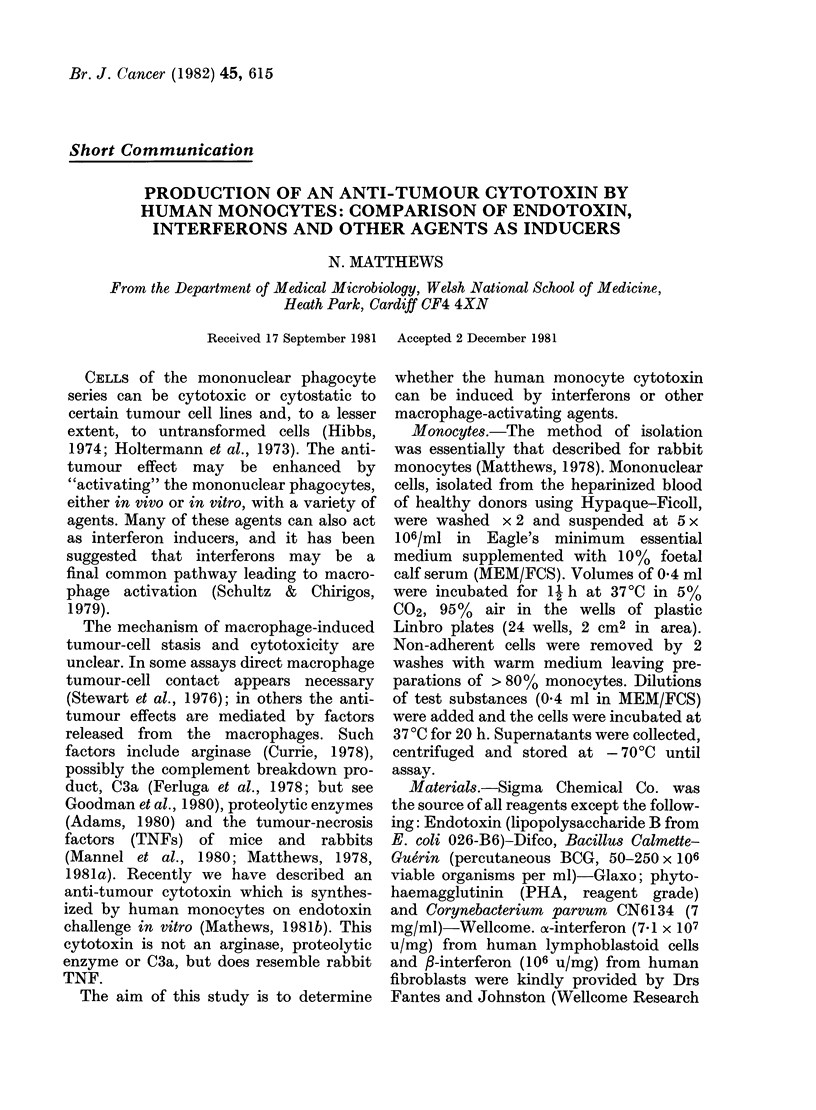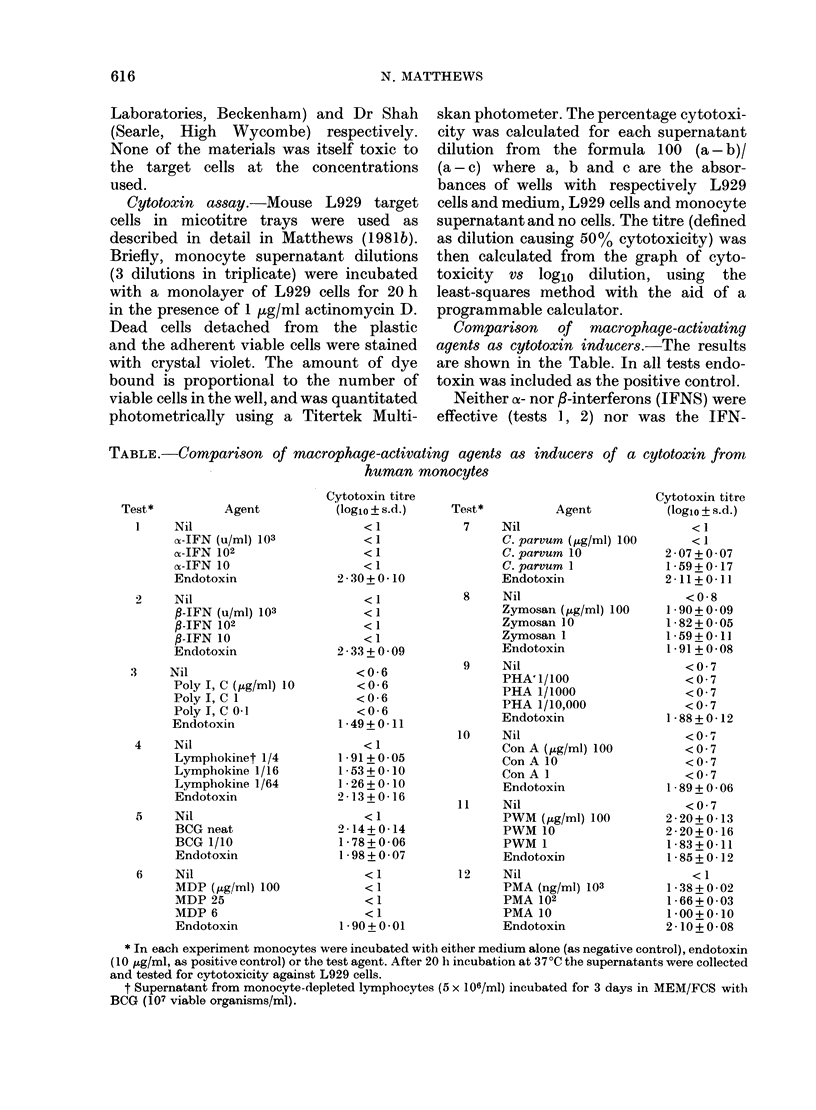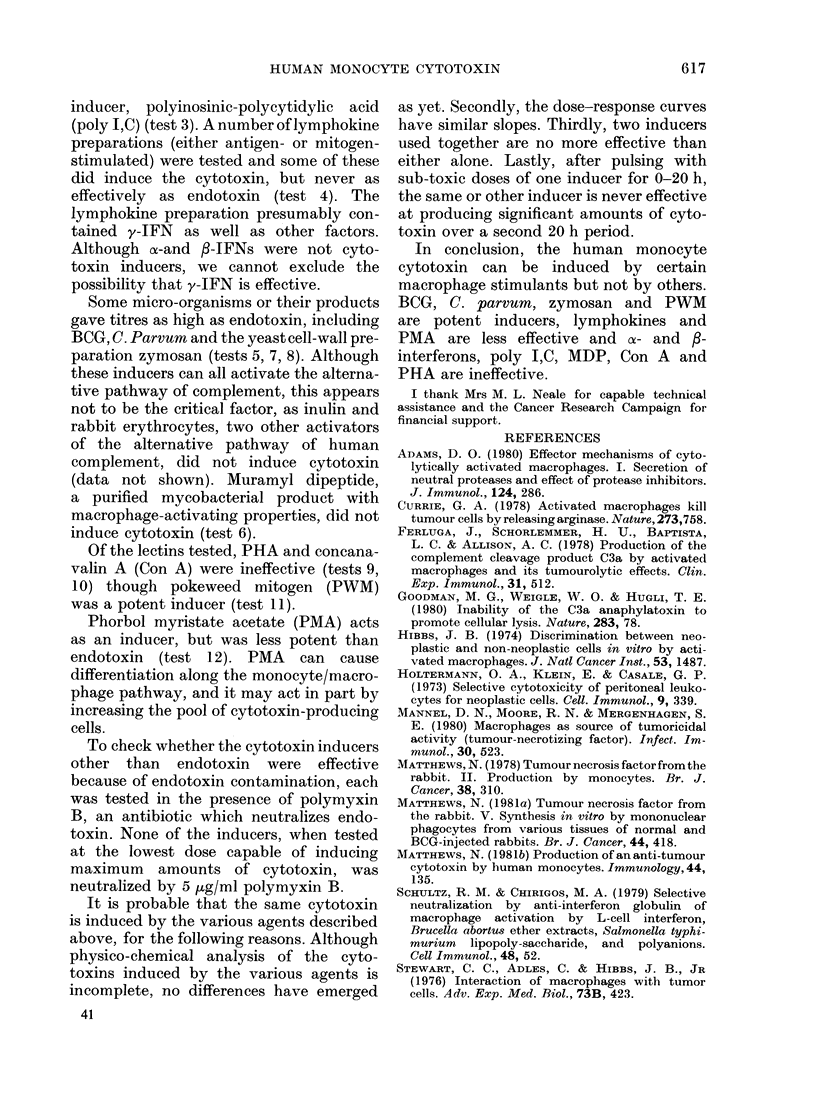# Production of an anti-tumour cytotoxin by human monocytes: comparison of endotoxin, interferons and other agents as inducers.

**DOI:** 10.1038/bjc.1982.99

**Published:** 1982-04

**Authors:** N. Matthews


Br. J. Cancer (1982) 45, 615

***Short Communication***

### PRODUCTION OF AN ANTI-TUMOUR CYTOTOXIN BY HUMAN MONOCYTES: COMPARISON OF ENDOTOXIN, INTERFERONS AND OTHER AGENTS AS INDUCERS

N. MATTHEWS

*From the Department of Medical Microbiology, Welsh National School of Medicine, Heath Park, Cardiff CF4 4XN*

Received 17 September 1981 Accepted 2 December 1981

CELLS of the mononuclear phagocyte series can be cytotoxic or cytostatic to certain tumour cell lines and, to a lesser extent, to untransformed cells (Hibbs, 1974; Holtermann *et al.*, 1973). The anti-tumour effect may be enhanced by "activating" the mononuclear phagocytes, either *in vivo* or *in vitro*, with a variety of agents. Many of these agents can also act as interferon inducers, and it has been suggested that interferons may be a final common pathway leading to macrophage activation (Schultz & Chirigos, 1979).

The mechanism of macrophage-induced tumour-cell stasis and cytotoxicity are unclear. In some assays direct macrophage tumour-cell contact appears necessary (Stewart *et al.*, 1976); in others the anti-tumour effects are mediated by factors released from the macrophages. Such factors include arginase (Currie, 1978), possibly the complement breakdown product, C3a (Ferluga *et al.*, 1978; but see Goodman *et al.*, 1980), proteolytic enzymes (Adams, 1980) and the tumour-necrosis factors (TNFs) of mice and rabbits (Mannel *et al.*, 1980; Matthews, 1978, 1981*a*). Recently we have described an anti-tumour cytotoxin which is synthesized by human monocytes on endotoxin challenge *in vitro* (Mathews, 1981*b*). This cytotoxin is not an arginase, proteolytic enzyme or C3a, but does resemble rabbit TNF.

The aim of this study is to determine whether the human monocyte cytotoxin can be induced by interferons or other macrophage-activating agents.

*Monocytes.*—The method of isolation was essentially that described for rabbit monocytes (Matthews, 1978). Mononuclear cells, isolated from the heparinized blood of healthy donors using Hypaque–Ficoll, were washed ×2 and suspended at $5 \times 10^6$/ml in Eagle's minimum essential medium supplemented with 10% foetal calf serum (MEM/FCS). Volumes of 0·4 ml were incubated for $1\frac{1}{2}$ h at 37°C in 5% $CO_2$, 95% air in the wells of plastic Linbro plates (24 wells, 2 cm$^2$ in area). Non-adherent cells were removed by 2 washes with warm medium leaving preparations of >80% monocytes. Dilutions of test substances (0·4 ml in MEM/FCS) were added and the cells were incubated at 37°C for 20 h. Supernatants were collected, centrifuged and stored at −70°C until assay.

*Materials.*—Sigma Chemical Co. was the source of all reagents except the following: Endotoxin (lipopolysaccharide B from *E. coli* 026-B6)–Difco, *Bacillus Calmette-Guérin* (percutaneous BCG, 50–250 × 10$^6$ viable organisms per ml)—Glaxo; phytohaemagglutinin (PHA, reagent grade) and *Corynebacterium parvum* CN6134 (7 mg/ml)—Wellcome. α-interferon ($7{\cdot}1 \times 10^7$ u/mg) from human lymphoblastoid cells and β-interferon (10$^6$ u/mg) from human fibroblasts were kindly provided by Drs Fantes and Johnston (Wellcome Research

Laboratories, Beckenham) and Dr Shah (Searle, High Wycombe) respectively. None of the materials was itself toxic to the target cells at the concentrations used.

*Cytotoxin assay.*—Mouse L929 target cells in micotitre trays were used as described in detail in Matthews (1981*b*). Briefly, monocyte supernatant dilutions (3 dilutions in triplicate) were incubated with a monolayer of L929 cells for 20 h in the presence of 1 μg/ml actinomycin D. Dead cells detached from the plastic and the adherent viable cells were stained with crystal violet. The amount of dye bound is proportional to the number of viable cells in the well, and was quantitated photometrically using a Titertek Multiskan photometer. The percentage cytotoxicity was calculated for each supernatant dilution from the formula 100 $(a-b)/(a-c)$ where a, b and c are the absorbances of wells with respectively L929 cells and medium, L929 cells and monocyte supernatant and no cells. The titre (defined as dilution causing 50% cytotoxicity) was then calculated from the graph of cytotoxicity *vs* $\log_{10}$ dilution, using the least-squares method with the aid of a programmable calculator.

*Comparison of macrophage-activating agents as cytotoxin inducers.*—The results are shown in the Table. In all tests endotoxin was included as the positive control.

Neither α- nor β-interferons (IFNS) were effective (tests 1, 2) nor was the IFN-

TABLE.—*Comparison of macrophage-activating agents as inducers of a cytotoxin from human monocytes*

| Test* | Agent | Cytotoxin titre ($\log_{10} \pm$ s.d.) | Test* | Agent | Cytotoxin titre ($\log_{10} \pm$ s.d.) |
|---|---|---|---|---|---|
| 1 | Nil | < 1 | 7 | Nil | < 1 |
| | α-IFN (u/ml) $10^3$ | < 1 | | *C. parvum* (μg/ml) 100 | < 1 |
| | α-IFN $10^2$ | < 1 | | *C. parvum* 10 | 2·07 ± 0·07 |
| | α-IFN 10 | < 1 | | *C. parvum* 1 | 1·59 ± 0·17 |
| | Endotoxin | 2·30 ± 0·10 | | Endotoxin | 2·11 ± 0·11 |
| 2 | Nil | < 1 | 8 | Nil | < 0·8 |
| | β-IFN (u/ml) $10^3$ | < 1 | | Zymosan (μg/ml) 100 | 1·90 ± 0·09 |
| | β-IFN $10^2$ | < 1 | | Zymosan 10 | 1·82 ± 0·05 |
| | β-IFN 10 | < 1 | | Zymosan 1 | 1·59 ± 0·11 |
| | Endotoxin | 2·33 ± 0·09 | | Endotoxin | 1·91 ± 0·08 |
| 3 | Nil | < 0·6 | 9 | Nil | < 0·7 |
| | Poly I, C (μg/ml) 10 | < 0·6 | | PHA 1/100 | < 0·7 |
| | Poly I, C 1 | < 0·6 | | PHA 1/1000 | < 0·7 |
| | Poly I, C 0·1 | < 0·6 | | PHA 1/10,000 | < 0·7 |
| | Endotoxin | 1·49 ± 0·11 | | Endotoxin | 1·88 ± 0·12 |
| 4 | Nil | < 1 | 10 | Nil | < 0·7 |
| | Lymphokine† 1/4 | 1·91 ± 0·05 | | Con A (μg/ml) 100 | < 0·7 |
| | Lymphokine 1/16 | 1·53 ± 0·10 | | Con A 10 | < 0·7 |
| | Lymphokine 1/64 | 1·26 ± 0·10 | | Con A 1 | < 0·7 |
| | Endotoxin | 2·13 ± 0·16 | | Endotoxin | 1·89 ± 0·06 |
| 5 | Nil | < 1 | 11 | Nil | < 0·7 |
| | BCG neat | 2·14 ± 0·14 | | PWM (μg/ml) 100 | 2·20 ± 0·13 |
| | BCG 1/10 | 1·78 ± 0·06 | | PWM 10 | 2·20 ± 0·16 |
| | Endotoxin | 1·98 ± 0·07 | | PWM 1 | 1·83 ± 0·11 |
| | | | | Endotoxin | 1·85 ± 0·12 |
| 6 | Nil | < 1 | 12 | Nil | < 1 |
| | MDP (μg/ml) 100 | < 1 | | PMA (ng/ml) $10^3$ | 1·38 ± 0·02 |
| | MDP 25 | < 1 | | PMA $10^2$ | 1·66 ± 0·03 |
| | MDP 6 | < 1 | | PMA 10 | 1·00 ± 0·10 |
| | Endotoxin | 1·90 ± 0·01 | | Endotoxin | 2·10 ± 0·08 |

* In each experiment monocytes were incubated with either medium alone (as negative control), endotoxin (10 μg/ml, as positive control) or the test agent. After 20 h incubation at 37 °C the supernatants were collected and tested for cytotoxicity against L929 cells.

† Supernatant from monocyte-depleted lymphocytes ($5 \times 10^6$/ml) incubated for 3 days in MEM/FCS with BCG ($10^7$ viable organisms/ml).

inducer, polyinosinic-polycytidylic acid (poly I,C) (test 3). A number of lymphokine preparations (either antigen- or mitogen-stimulated) were tested and some of these did induce the cytotoxin, but never as effectively as endotoxin (test 4). The lymphokine preparation presumably contained $\gamma$-IFN as well as other factors. Although $\alpha$-and $\beta$-IFNs were not cytotoxin inducers, we cannot exclude the possibility that $\gamma$-IFN is effective.

Some micro-organisms or their products gave titres as high as endotoxin, including BCG, *C. Parvum* and the yeast cell-wall preparation zymosan (tests 5, 7, 8). Although these inducers can all activate the alternative pathway of complement, this appears not to be the critical factor, as inulin and rabbit erythrocytes, two other activators of the alternative pathway of human complement, did not induce cytotoxin (data not shown). Muramyl dipeptide, a purified mycobacterial product with macrophage-activating properties, did not induce cytotoxin (test 6).

Of the lectins tested, PHA and concanavalin A (Con A) were ineffective (tests 9, 10) though pokeweed mitogen (PWM) was a potent inducer (test 11).

Phorbol myristate acetate (PMA) acts as an inducer, but was less potent than endotoxin (test 12). PMA can cause differentiation along the monocyte/macrophage pathway, and it may act in part by increasing the pool of cytotoxin-producing cells.

To check whether the cytotoxin inducers other than endotoxin were effective because of endotoxin contamination, each was tested in the presence of polymyxin B, an antibiotic which neutralizes endotoxin. None of the inducers, when tested at the lowest dose capable of inducing maximum amounts of cytotoxin, was neutralized by 5 $\mu$g/ml polymyxin B.

It is probable that the same cytotoxin is induced by the various agents described above, for the following reasons. Although physico-chemical analysis of the cytotoxins induced by the various agents is incomplete, no differences have emerged as yet. Secondly, the dose–response curves have similar slopes. Thirdly, two inducers used together are no more effective than either alone. Lastly, after pulsing with sub-toxic doses of one inducer for 0–20 h, the same or other inducer is never effective at producing significant amounts of cytotoxin over a second 20 h period.

In conclusion, the human monocyte cytotoxin can be induced by certain macrophage stimulants but not by others. BCG, *C. parvum*, zymosan and PWM are potent inducers, lymphokines and PMA are less effective and $\alpha$- and $\beta$-interferons, poly I,C, MDP, Con A and PHA are ineffective.

I thank Mrs M. L. Neale for capable technical assistance and the Cancer Research Campaign for financial support.

### REFERENCES

Adams, D. O. (1980) Effector mechanisms of cytolytically activated macrophages. I. Secretion of neutral proteases and effect of protease inhibitors. *J. Immunol.*, **124**, 286.

Currie, G. A. (1978) Activated macrophages kill tumour cells by releasing arginase. *Nature*, **273**, 758.

Ferluga, J., Schorlemmer, H. U., Baptista, L. C. & Allison, A. C. (1978) Production of the complement cleavage product C3a by activated macrophages and its tumourolytic effects. *Clin. Exp. Immunol.*, **31**, 512.

Goodman, M. G., Weigle, W. O. & Hugli, T. E. (1980) Inability of the C3a anaphylatoxin to promote cellular lysis. *Nature*, **283**, 78.

Hibbs, J. B. (1974) Discrimination between neoplastic and non-neoplastic cells *in vitro* by activated macrophages. *J. Natl Cancer Inst.*, **53**, 1487.

Holtermann, O. A., Klein, E. & Casale, G. P. (1973) Selective cytotoxicity of peritoneal leukocytes for neoplastic cells. *Cell. Immunol.*, **9**, 339.

Mannel, D. N., Moore, R. N. & Mergenhagen, S. E. (1980) Macrophages as source of tumoricidal activity (tumour-necrotizing factor). *Infect. Immunol.*, **30**, 523.

Matthews, N. (1978) Tumour necrosis factor from the rabbit. II. Production by monocytes. *Br. J. Cancer*, **38**, 310.

Matthews, N. (1981*a*) Tumour necrosis factor from the rabbit. V. Synthesis *in vitro* by mononuclear phagocytes from various tissues of normal and BCG-injected rabbits. *Br. J. Cancer*, **44**, 418.

Matthews, N. (1981*b*) Production of an anti-tumour cytotoxin by human monocytes. *Immunology*, **44**, 135.

Schultz, R. M. & Chirigos, M. A. (1979) Selective neutralization by anti-interferon globulin of macrophage activation by L-cell interferon, *Brucella abortus* ether extracts, *Salmonella typhimurium* lipopoly-saccharide, and polyanions. *Cell Immunol.*, **48**, 52.

Stewart, C. C., Adles, C. & Hibbs, J. B., Jr (1976) Interaction of macrophages with tumor cells. *Adv. Exp. Med. Biol.*, **73B**, 423.